# Opportunistic screening for osteoporosis using hydroxyapatite measurements of the vertebral by thorax dual-energy spectral CT in postmenopausal females

**DOI:** 10.1038/s41598-022-26237-4

**Published:** 2022-12-14

**Authors:** Lei Deng, Yue Yao, A.-Li Shang, Tongtong Du, Jingbin Zhang, Quanxin Yang, Jianying Li, Qian Wang, Xiaohui Li

**Affiliations:** 1 Department of Radiology, the Second Affiliated Hospital of Xi’an Jiaotong University, #157, Xi Wu Road, Xi’an, 710004 Shaanxi China; 2 GE Healthcare, Computed Tomography Research Center, 1South Tongji Road, Beijing, 100176 China; 3 Department of Health Management, the Second Affiliated Hospital of Xi’an Jiaotong University, #5, Jian Qiang Road, Xi’an, 710016 Shaanxi China

**Keywords:** Endocrine system and metabolic diseases, Osteoporosis

## Abstract

The purpose of this study was to evaluate the feasibility of opportunistic screening for osteoporosis in postmenopausal females using the dual-energy CT(DECT)-derived hydroxyapatite (HAP) concentration and CT value of L1-vertebra. 239 consecutive postmenopausal female patients were enrolled and underwent both chest DECT and Dual energy X-ray absorptiometry (DXA). According to the T-score of the 1st lumbar vertebra on DXA, patients were divided into the osteoporosis group (T $\le$− 2.5, n = 112) and non-osteoporosis group (T $>$ − 2.5, n = 127). The HAP values of the 1st lumbar vertebra were measured from the coronal-view HAP(Fat)-based material decomposition(MD) images, and CT values were measured on the 75 keV monochromatic image. The cutoff values of using HAP and CT value for diagnosing osteoporosis were obtained by drawing receiver operating characteristic (ROC) curves. Both HAP and CT value of the 1st lumbar vertebra had moderate-high correlation with bone-mineral-density measurement on DXA (HAP, r = 0.614; CT value, r = 0.625; all *p* < 0.01). The area-under-the-curve (AUC), sensitivity, specificity, PPV and NPV for diagnosing osteoporosis was 0.754, 0.714, 0.693, 0.68 and 0.752 using HAP (cutoff value: 142.05 mg/cm^3^) and 0.766, 0.741, 0.7, 0.685 and 0.754 using CT value (cutoff value: 132HU), respectively. HAP measurements on HAP(Fat)-based MD images in DECT could provide reasonably accurate BMD quantification for diagnosing osteoporosis in postmenopausal females. DECT prescribed for lung cancer screening could also provide opportunistic screening for osteoporosis, extending the clinical application of DECT without additional radiation to patients.

## Introduction

Osteoporosis is a frequent metabolic bone disorder in the postmenopausal women and in the elderly, characterized by decrease of bone mineral density (BMD) and microarchitectural deterioration of bone tissue, with a consequent increase in bone fragility and susceptibility to fracture^1^.

Due to aging of the population, the number of women in the postmenopausal period has increased, especially those in 50 years of age or older^2^. And women spend approximately a third of their lives in their postmenopausal years and estrogen deficiency is a major factor contributing to bone mass reduction and structural deterioration^3^. Since the deterioration of BMD can be slowed down and prevented by lifestyle measures and drug therapy, early diagnosis and treatment are essential to prevent osteoporosis and effectively reduce the incidence of fractures and other complications^4,5^.

Dual-energy X-ray absorptiometry (DXA) is widely recognized as the reference standard for diagnosing osteoporosis^6^. However, various limitations of DXA have been described such as inability to assess the necessary particulars of the bone marrow composition and distortion of estimated bone mass values caused by overlying abdominal tissue, vascular calcifications, bowel contents and degenerative spine changes, leading to bias in diagnosis^7,8^. It is a common knowledge that Quantitative Computed Tomography (QCT) has been established for volumetric BMD assessment. However, the composition of trabecular bone, the varying water or fat content in calibration phantoms could have impact on the accuracy of QCT, which is obvious in the conventional single-kVp scan^9,10^. And multiple levels of phantom or angular correction region-of-interest (ROI) measurements are required for calibrating bone, muscle, and fat. Therefore, the application of QCT in assessing osteoporosis is still limited.

Multiple studies have found that the use of trabecular Hounsfield units (HU) to measure BMD during regularly performed CT examinations can be used to the opportunistic assessment and screening for osteoporosis with promising results^11,12^. Nevertheless, the inhomogeneous composition of trabecular bone consisting of bone minerals, collagen matrix, water, bone marrow and fat content is likely to cause inaccuracy of HU analysis in order to assess osteoporosis^13–16^. Initial studies involving early dual-energy CT(DECT) concepts for BMD evaluation were published more than 2 decades ago^17^. Compared to conventional CT, DECT allows for both material decomposition for more accurate material separation and monochromatic image sets for less beam hardening artifacts in the images by utilizing energy dependence of the photoelectric effect at different x-ray spectra and this technique has provided novel clinically relevant information regarding various musculoskeletal (MSK) applications^17–20^. The material decomposition (MD) technique in DECT allows for BMD measurements with minimal influence of bone marrow fat.

Opportunistic screening for diseases has emerged widely in the field. Chest CT examination is the most commonly used CT examination in clinical work. Retrieval of BMD information available on chest CT requires no additional cost, patient time and radiation exposure, and the BMD obtained by chest CT can be used for the analysis of osteoporosis. Since DECT generates both MD images and monochromatic image sets in a single CT scan, in this study we aimed to evaluate the feasibility of opportunistic screening for osteoporosis in post menopausal females using the hydroxyapatite (HAP) concentration measurements and CT value measurements on the thorax DECT images of the first lumbar vertebra, using DXA as a standard reference.

## Materials and methods

### Study population

Our institutional review board (the Second Affiliated Hospital of Xi’an Jiaotong University committee) approved this prospective study. All methods were performed in accordance with the relevant guidelines and regulations of the Second Affiliated Hospital of Xi’an Jiaotong University committee. All study participants provided informed consent.

**Figure 1:**
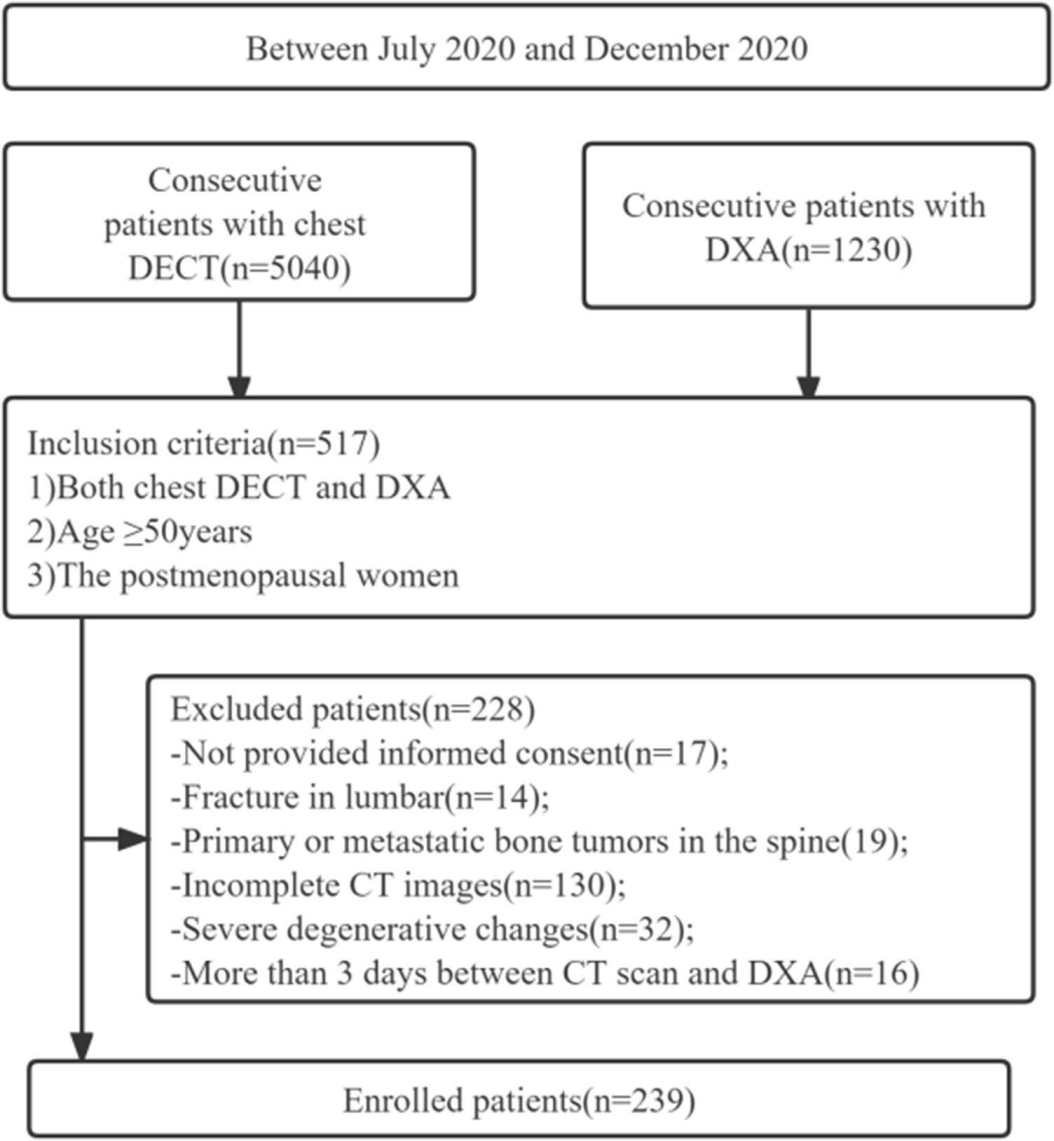
Flowchart of patient selection. *DECT:* dual energy CT, *DXA:* dual-energy X-ray absorptiometry.

A total of 239 postmenopausal female patients were prospectively enrolled consecutively between July 2020 and December 2020. All patients underwent both chest DECT scan and lumbar spine DXA. Patients were excluded if they had the following conditions: (a) no informed consent; (b) fracture in lumbar; (c) primary or metastatic bone tumors in the spine; (d) incomplete CT images; (f) Severe degenerative changes; (g) more than 3 days between CT scan and DXA (Fig. 1).

### Imaging protocol

Lumbar DXA was used as standard reference which was performed on a Lunar Prodigy Advance bone densitometer (GE Healthcare, Little Chalfont, UK). For each vertebra, the manufacturer software automatically calculated BMD values and standardized T-scores and Z-scores according to World Health Organization (WHO) guidelines. The diagnosis of osteoporosis and osteopenia was based on the lowest reported DXA-derived T-score of the lumbar spine. But in this study, we chose L1 vertebra BMD values for comparison. Patients were categorized as having osteoporosis (T-score $\le$ − 2.5), osteopenia or normal BMD (T-score $> -$2.5) according to WHO criteria^6^.

The chest DECT was performed on a 256-row Revolution CT scanner (GE Healthcare, USA) using the spectral imaging mode: 80-140kVp fast switching; 200 mA; rotation speed, 0.5 s/r to result in 100mAs; helical scan pitch, 0.992:1. None of the patients received contrast media.

### CT image reconstruction

All the CT images were transmitted to an AW4.7 workstation (GE Healthcare, Milwaukee, WI USA). All images were displayed on a coronal-view using a bone window and bone reconstruction kernel; reconstruction slice thickness/increment was 0.625 mm; the slice thickness on the AW4.7 was 1.25 mm for image analysis purpose. The HAP values of the 1st lumbar vertebra were measured on the HAP(Fat)-based MD images by using the material decomposition (MD) software, and CT values of the 1st lumbar vertebra were measured on the 75 keV monochromatic images by using the monochromatic (Mono +) software. The region-of-interest (ROI) was selected as large as possible, avoiding the vertebral cortex, dense bone islands, venous plexus, or focal lesions including compression fractures. The copy-and-paste function was used for placing ROI between the HAP(Fat)-based MD images and 75 keV monochromatic images to reduce inconsistency. The manual positioning of the single ROI was repeated for 3 times. The final data being recorded were mean values averaged from the 3 measurements.

### Statistical analysis

The correlations between DXA-derived BMD value and DECT-derived HAP value and CT value were performed by using Pearson Correlation. The HAP values or CT values of osteoporotic and non-osteoporotic groups were compared by using the independent sample t-test. A regression analysis was performed to clarify the correlation between HAP concentration and CT value. A two-tailed *p*-value of $<$ 0.05 was considered statistically significant. The cut-off for using HAP value or CT value to diagnose osteoporosis was obtained by drawing receiver operating characteristic (ROC) curves. The sensitivity, specificity, positive predictive value (PPV), and negative predictive value (NPV) were calculated and represented with 95% confidence intervals. All statistical analyses were performed using SPSS statistical software (version 22.0, IBM SPSS Statistics).

## Results

### Quantitative analysis

Of the 239 patients, a total of 112 patients were diagnosed as osteoporotic, and 127 cases were diagnosed as non-osteoporotic according to the BMD measurement on DXA. Both HAP value and CT value of the 1st lumbar vertebra had moderate-high correlation with BMD on DXA (HAP value, r = 0.614; CT value, r = 0.625; all *p* < 0.05).

The mean BMD and T-score on DXA images were 0.63 g/cm^2^ and − 3.2 for the osteoporosis group and 0.84 g/cm^2^ and − 1.5 for the non-osteoporosis group, respectively. There was significant difference between the osteoporosis and non-osteoporosis groups in BMD and T-score (all *p* $<$ 0.001). The mean HAP value and CT value on DECT images of the osteoporosis group were 128 mg/cm^3^ and 113.81 HU, respectively which was significantly lower than the corresponding value of the non-osteoporosis group (HAP, 153 mg/cm^3^; CT value, 150.09HU; all *p* $<$ 0.001) (Table 1).

**Table 1:** Characterization of the patient population in this study.

Characteristics		Osteoporosis	Non-osteoporosis	*p-*value
Mean age, (range)		60.51 ± 6.94, (50–82)	56.64 ± 5.88, (50–79)	
Number of patients		112	127	
BMI		24.24 ± 2.95	24.55 ± 3.27	> 0.05
DXA	BMD (g/cm^2^)	0.63 ± 0.09	0.84 ± 0.08	< 0.001
	T-score	− 3.2 ± 0.56	− 1.5 ± 0.75	< 0.001
DECT	HAP(fat) (mg/cm^3^)	128 ± 23.34	153 ± 27.46	< 0.001
	75 keV CT-value (HU)	113.81 ± 33.87	150.09 ± 38.48	< 0.001

Age was given in years. BMI: body mass index (kg/m^2^).

### Diagnostic performance using HAP and CT value in DECT

**Figure 2:**
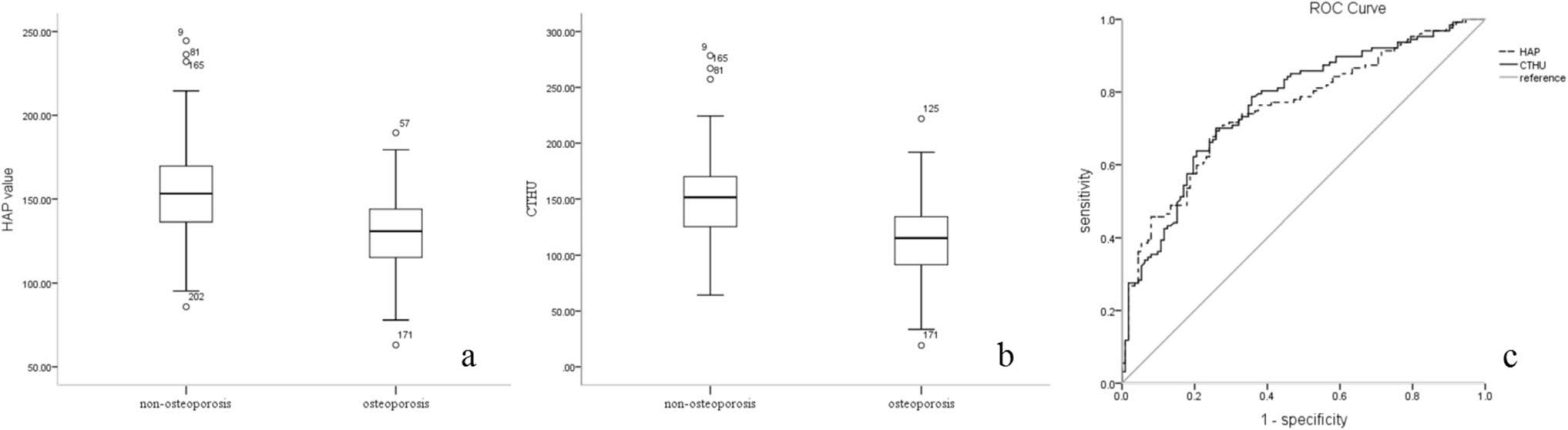
Diagnosing osteoporosis by using HAP concentration and CT value. Box chart. There was significant difference between the osteoporosis group and non-osteoporosis group (**a**): HAP concentration; (**b**): CT value. (**c**): ROC of using HAP concentration (dotted line) and Hounsfield unit (HU) measurements (solid line) for the detection of osteoporosis. The area-under-the-curve (AUC) using HAP and CT value was 0.754 and 0.766, respectively for diagnosing osteoporosis.

**Figure 3:**
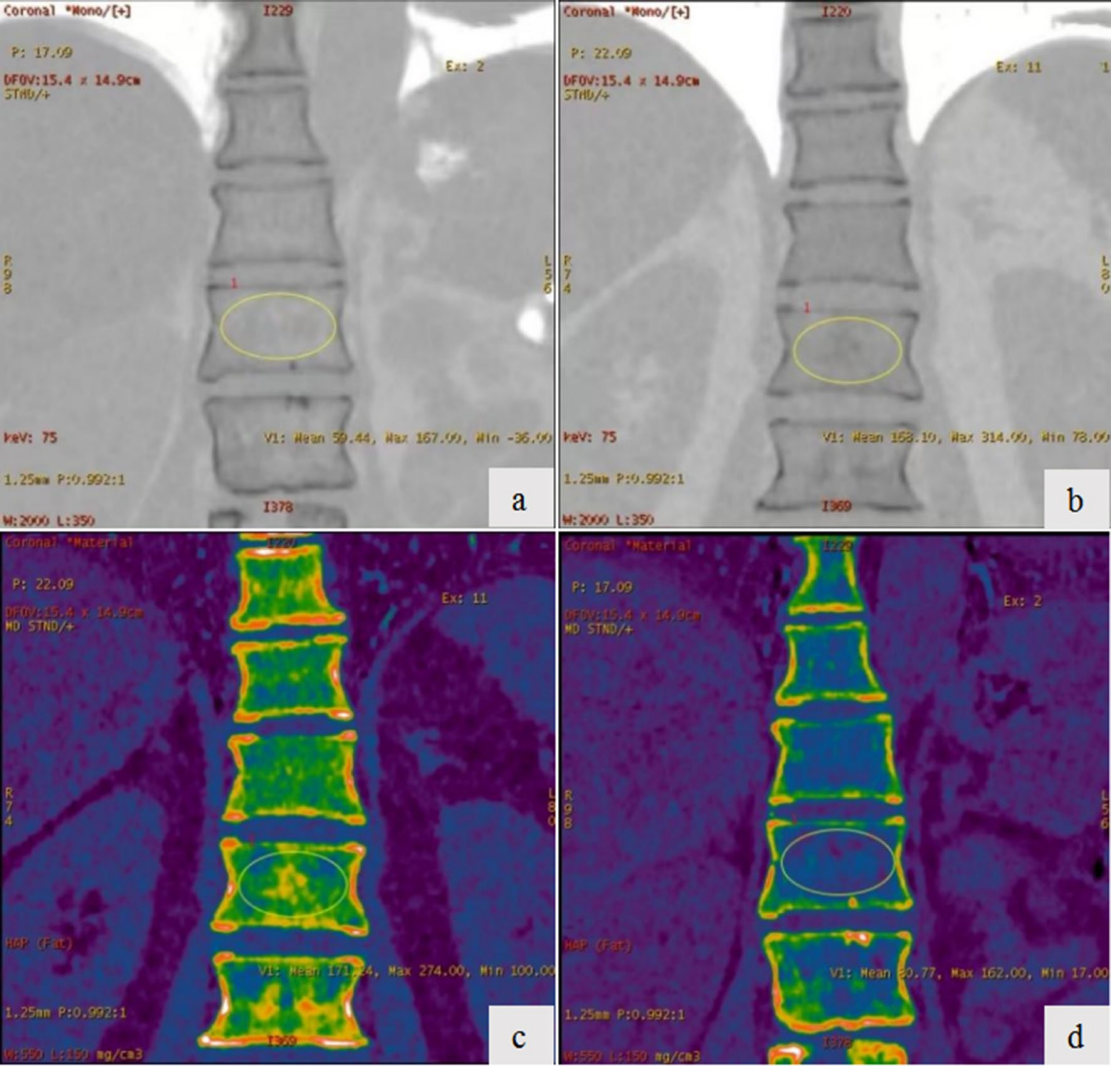
The coronal-view CT image and HAP image of thoracolumbar spine. The ROI was drawn on the 1st lumbar. CT value: (**a**), Non-Osteoporosis group; (**b**), osteoporosis group; HAP concentration: (**c**), Non-Osteoporosis group; (**d**), osteoporosis group.

**Figure 4:**
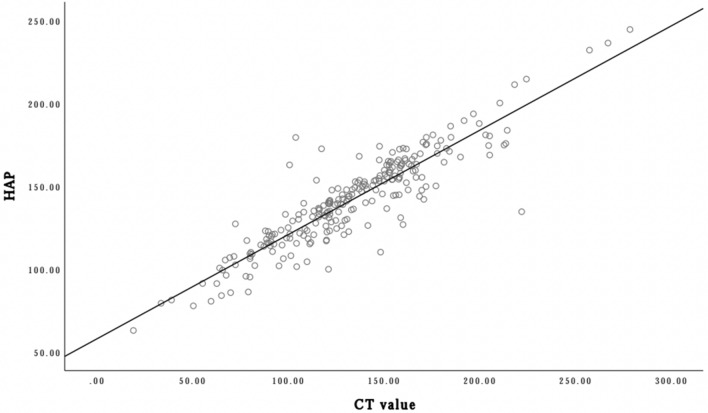
Scatter plots of CT value and HAP. There was a linear correlation between CT value and HAP. And the linear regression equation was: $HAP=57.941+0.629\times CT value$ (black slant line).

The HAP cutoff value for the 1st lumbar vertebra for diagnosing osteoporosis was 142.055 mg/cm^3^. The sensitivity, specificity, PPV and NPV were 0.714, 0.693, 0.680 and 0.752, respectively. The area under the ROC curve (AUC) was 0.754. The CT cutoff value for the 1st lumbar vertebra for diagnosing osteoporosis was 132HU. The sensitivity, specificity, PPV, NPV and AUC were 0.741, 0.7, 0.685, 0.754 and 0.766, respectively (Table 2, Fig. 2, 3). The regression analysis showed that HAP measurement was correlated with CT value, and there was a linear correlation between them (R^2^ = 0.815, *P* $<0.001$). The linear regression equation was: $HAP=57.941+0.629\times CT value$ (Fig. 4).

**Table 2:** The performance of HAP concentration and CT value in DECT in diagnosing osteoporosis.

	Cut off	AUC	Sensitivity	Specificity	PPV	NPV
HAP value	142.05 mg/cm^3^	0.754	0.714	0.693	0.680	0.752
CT value	132HU	0.766	0.741	0.700	0.685	0.754

HAP and CT-value assessment derived from DECT both showed high diagnostic accuracy for the detection of osteoporosis with a significant difference regarding the overall AUC.

*AUC:* area under the curve, *NPV:* negative predictive value, *PPV:* positive predictive value.

## Discussion

In our study, we compared the performance of diagnosing osteoporosis using HAP concentration measurement on MD images and CT value on monochromatic images in DECT with DXA as standard reference. The results of this study demonstrated that HAP measurement on HAP (Fat)-based MD images in DECT showed a similar good performance as the CT value measurement on monochromatic images in diagnosing osteoporosis in middle-aged and elder females. Our study suggested that DECT prescribed for lung cancer screening could also provide opportunistic screening for osteoporosis, extending the clinical application of DECT without additional radiation to patients.

DECT could achieve material separation and generate different base material pairs according to clinical needs and a set of monochromatic images at the same time in a single scan. Some experimental and clinical studies have demonstrated that the base substance density value can reflect the material content if the composition of the selected base material pairs is close to the actual material^21,22^. Dong Yue et al.^23^ studied the selection of calcium (water) substance pairs, indicating that calcium (water) density in the vertebral body decreases with age. There are complex components in cancellous bone that contain red marrow, yellow marrow, fat, water, and bone minerals, so the Ca(Wa) density obtained from the DECT-determined measurements might be affected by these factors, especially when the fat content of the cancellous bone increases with age. Therefore, HAP (FAT) was selected in this study, and it was found that HAP concentration using HAP(Fat) material pair was also strongly correlated with BMD. And the CT value (HU) was also measured on the DECT monochromatic images that are less susceptible to beam hardening artifacts.

Pickhardt et al. utilized ROC curve analysis to derive optimal cutoff values using CT value measurement in conventional CT for the diagnosis and exclusion of osteoporosis^11^. They proposed a threshold of HU ≤ 135 in the L1st for diagnosing osteoporosis with sensitivity, specificity, PPV and NPV of 75.5%, 75.4%, 47.2% and 91.3%, respectively. The correlation between bone density and fracture risk is well established with subsequent studies showing good correlations between HU measurements and bone density^24,25^. Since osteoporosis is a risk factor for future fractures, it is important to identify patients with osteoporosis earlier. Similar to Pickhardt et al. our current results indicated a cutoff value of HU ≤ 132 for CT value measurement using the 75 keV monochromatic images for the diagnosis of osteoporosis which yielded sensitivity, specificity, PPV and NPV of 74.1%, 70.0%, 68.5% and 75.4%, respectively. Our findings suggested that screening results for women over 50 years were consistent with previous studies.

Lee et al. compared CT-derived HU to DXA in the lumbar spine using the transverse and sagittal images and demonstrated that transverse and sagittal HU measurements were in agreement to each other^12^. In our study, we used coronal plane for image analysis and ROI placement, because BMD was also evaluated in the coronal position of the lumbar spine by DXA, so as to maintain the consistency of position and ensure the matching degree of data. At the same time, we emphasized measuring L1 lumbar vertebra in our study for several reasons. Firstly, as indicated by Pickhardt et al.^11^, using L1 provided the same or even better results than multiple vertebral bodies in assessing osteoporosis. Secondly, L1 level is easy to identify, which improve the efficiency and reproducibility. It is included on all the standard chest and abdominal CT scan, can potentially substantially increase the rate of large scale of osteoporosis screening.

In this study, we found that the HAP density value and CT value in DECT showed no significantly different performance in diagnosing osteoporosis. DECT produces two very important types of images: a set of monochromatic images (keV images) and material-decomposition images (HAP images). In theory, the material-specific MD images are more advantageous in BMD measurements because they are less susceptible to beam hardening artifacts and iodine contamination. On the other hand, previous studies also indicated that monochromatic images in DECT are also less susceptible to beam hardening artifacts than the polychromatic images. Since in our study, no iodine contrast agent was used, and the CT numbers were extracted from the 75 keV monochromatic images. The similar performance between the use of HAP density value and 75 keV CT value should not be a surprise to us. The similar diagnostic accuracy results indicated that in the case where CT scan was performed without iodine contrast, the CT values obtained from the monochromatic images performed as well as the more sophisticated material-decomposition based measurements. So, the results provided evidence that one could just use the CT value in DECT to reduce workload for radiologists. As we indicated earlier, DECT is being used more often clinically for cancer screening at reasonable radiation dose levels. Our study showed the additional clinical values that the existing DECT images could provide and that the simple CT value from the monochromatic images could be used for the screening of osteoporosis. Since monochromatic image is one of the important features in DECT and is more familiar to clinicians, the realization of the added-value of DECT in osteoporosis screening with parameters that are in theory less susceptible to beam hardening should not significantly increase the workload for clinicians.

Our study has the following limitations: (1) We used DXA as the reference standards in our study duo to the lack of QCT. DXA suffers from tissue overlaps (by overlying abdominal tissue, vascular calcifications, bowel contents and degenerative spine changes), results in higher degrees of measurement variation to reflect the true BMD values, which may negatively impact our results and weaken the conclusions of our study. (2) In our study, in order to reduce potential radiation risks to patients, we did not scan patient twice (with both DECT and conventional 120kVp) to directly compare the performance of diagnosing osteoporosis between the monochromatic images in DECT and the 120kVp images in the convential CT. Therefore, our study could not show the superiority of DECT over conventional CT. The comparison between monochromatic images (with DECT) and polychromatic images (with single tube voltage CT) needs to be carried out using better reference standards such as values from QCT in the future. (3) Fracture risk prediction and osteoporosis treatment decisions are often determined by DXA-based hip T-scores. Since we used the influence information from chest CT, our analysis was based on vertebral measurements only. Its application for fracture risk prediction needs further investigation. (4) Clinical risk factors for bone mineral density loss were not evaluated in this study. Since we only studied a population under routine health examination, only a small percentage of the population was affected by external factors (drugs, other diseases). (5) We did not formally evaluate the potential benefits and costs of diagnosing osteoporosis using the CT threshold we identified, but we speculate that substantial medical savings could be achieved by providing additional osteoporosis detection with chest CT, followed by appropriate treatment to reduce fracture risk, and a reduction in the number of normal DXA studies.

In conclusion, this study demonstrated HAP measurements on HAP (Fat)-based MD images in DECT could provide reasonably accurate BMD quantification in diagnosing osteoporosis for middle-aged and elder female patients who must perform DECT. This study also demonstrated that while chest DECT is primarily designed to detect and diagnose diseases in the lungs, it may also provide opportunistic screening for osteoporosis at the same time using the same images, extending the clinical application of DECT without the need for additional imaging, radiation exposure, cost of equipment or patient time.

## Supplementary Information

Supplementary Information.

## Data Availability

All data generated or analysed during this study are included in this published article [and its Supplementary Information files].
